# Pollen Developmental Arrest: Maintaining Pollen Fertility in a World With a Changing Climate

**DOI:** 10.3389/fpls.2019.00679

**Published:** 2019-05-24

**Authors:** Ettore Pacini, Rudy Dolferus

**Affiliations:** ^1^Department of Life Sciences, University of Siena, Siena, Italy; ^2^Agriculture and Food, Commonwealth Scientific and Industrial Research Organisation, Canberra, ACT, Australia

**Keywords:** pollen, developmental arrest, desiccation, viability, fertility, abiotic stress tolerance

## Abstract

During evolution of land plants, the haploid gametophytic stage has been strongly reduced in size and the diploid sporophytic phase has become the dominant growth form. Both male and female gametophytes are parasitic to the sporophyte and reside in separate parts of the flower located either on the same plant or on different plants. For fertilization to occur, bi-cellular or tri-cellular male gametophytes (pollen grains) have to travel to the immobile female gametophyte in the ovary. To survive exposure to a hostile atmosphere, pollen grains are thought to enter a state of complete or partial developmental arrest (DA). DA in pollen is strongly associated with acquisition of desiccation tolerance (DT) to extend pollen viability during air travel, but occurrence of DA in pollen is both species-dependent and at the same time strongly dependent on the reigning environmental conditions at the time of dispersal. Several environmental stresses (heat, drought, cold, humidity) are known to affect pollen production and viability. Climate change is also posing a serious threat to plant reproductive behavior and crop productivity. It is therefore timely to gain a better understanding of how DA and pollen viability are controlled in plants and how pollen viability can be protected to secure crop yields in a changing environment. Here, we provide an overview of how DA and pollen viability are controlled and how the environment affects them. We make emphasis on what is known and areas where a deeper understanding is needed.

## Introduction

During evolution, land plants evolved from aquatic green algae (Charophytes) to colonize the Earth’s land mass. To survive on land, plants were required to undergo dramatic morphological and physiological changes to adapt to a hostile atmosphere. This move requested significant changes to reproductive strategies in particular. Early land plants (hornworts, liverworts, mosses and ferns) colonized areas with easy access to water; reproduction was still water-dependent, requiring a film of water on the plant surface. The flagellate male gametes had to actively disperse for fertilization to occur. To conquer the rest of the landmass plants had to evolve mechanisms to control transpiration and actively acquire water from the soil (root system, vasculature and stomata). But importantly, there was a requirement for reproduction to occur under atmospheric conditions and independent of water (Yin-Long et al., 2012). In angiosperms and gymnosperms, the diploid sporophytic generation became dominant and the haploid gametophytes became parasitic and physically part of the sporophyte. Similarly to many animals, sexual reproduction in higher land plants requires internal fertilization. The sporophyte is immobile and different mechanisms have evolved to pass on genetic information to offspring.

**Figure 1:**
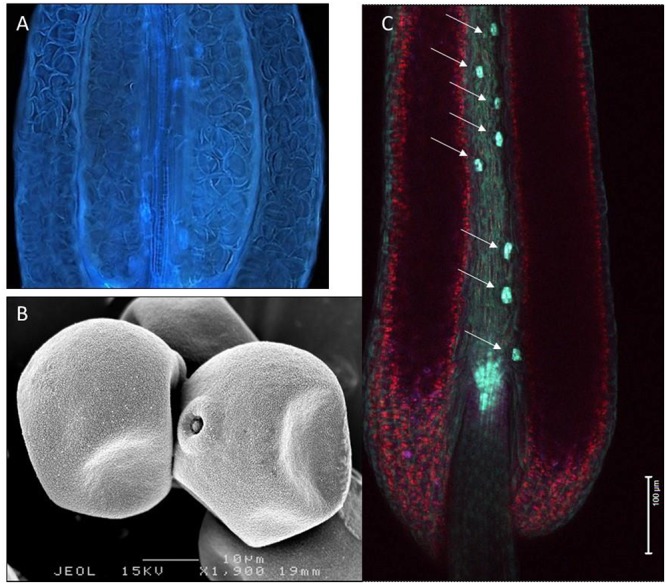
Variation in hydration levels of recalcitrant rice pollen (*Oryza sativa*) prior to anthesis. **(A)** Side view of a water-stressed rice anther (UV lighting) shows the presence of many collapsed pollen grains. **(B)** Cryo-SEM picture showing partial dehydration in anthesis stage rice pollen. **(C)** Expression of the rice *OsNCED3* ABA biosynthetic gene in the guard cells of stomata located on the anther connective tissue (arrows). Anther stomata were shown to play a role in regulating anther and pollen dehydration.

Exposure of pollen grains to the environment is a critical step in the reproductive cycle of higher plants. Adverse environmental conditions affect all stages of male gametophyte development (Mesihovic et al., 2016). Because pollen development occurs inside the anther and flowers of the mother plant, negative effects of the environment on the sporophyte are also communicated to the gametophytes (Hinojosa et al., 2018). Anther and pollen desiccation are crucial to prepare pollen for dispersal when environmental conditions are optimal for pollen survival. When the mother plant experiences stress conditions, this signal will reach the pollen grains and affect preparation and timing of pollen dispersal. Release of pollen in the environment is therefore controlled by an equilibrium between the physiological state of the sporophyte and the atmospheric conditions (Begcy and Dresselhaus, 2018). However, when pollen grains are dispersed, they need to autonomously respond and adapt to environmental fluctuations. In order to survive, they must activate an homeostatic mechanism to maintain turgor pressure as constant as possible to protect cellular functions and maintain viability. After landing on the stigma, pollen emits the pollen tube, which grows toward the female gamete inside the ovule where fertilization occurs (Knox et al., 1993; Bateman and Dimichele, 1994). At anthesis, pollen grains of some plants enter a metabolically “inactive state” to support survival during pollen dispersal. When acquiring DA, pollen grains lose water and reach a state of complete or partial desiccation tolerance (DT) – depending on environmental conditions. There is a high degree of morphological and physiological diversity in plant pollen at the time of dispersal (see summary in Table 1). Plant pollen can be classified as recalcitrant or orthodox depending on the water content at pollen dispersal (Table 1; Franchi et al., 2011). DA occurs in plants producing both orthodox and recalcitrant pollen. The two pollen types differ in the percentage of cellular water, accumulation of biochemical components and morphology of pollen at the time of dispersal (Pacini and Dolferus, 2016). In both pollen types, viability depends on the degree of dehydration and the level of DT and DA and this is heavily influenced by environmental conditions such as temperature and humidity. Some self-pollinating plants produce recalcitrant pollen grains which are normally dispersed in a highly hydrated stage, which do not enter DA and are very short-lived (e.g., rice and wheat). However, partial dehydration of recalcitrant pollen can occur when the mother plant experiences water stress (Figure 1) and this can further affect pollen viability and reproductive success (Turner, 1993; Bots and Mariani, 2005; Franchi et al., 2011; Firon et al., 2012). We know little about the genetic control of DA in plants producing orthodox pollen, nor do we know whether plants producing recalcitrant pollen have the ability to desiccate and enter a state of DA to extend pollen longevity.

**Table 1:** Classification of ripe pollen biodiversity according to eco- and cyto-physiological features, and examples of some representative plant species.

Pollen type at presentation	Starch content	Pollen type	
		Two-celled	Three-celled
**Orthodox pollen:**	Starchy	- *Olea europaea* (Oleaceae): PK, A (2)	- *Wolffia arrhiza* (Araceae): PK, Z (1)
- Desiccation tolerant		- *Erica arborea* (Ericaceae): tetrad pollen, PK, Z, A (2)	- *Lilium bienne* (Liliaceae): PK, Z (2)
- [H_2_O] dispersal: <30%		- *Atropa belladonna* (Solanaceae): PK, Z (1)	- *Nelumbo nucifera* (Nelumbonaceae): PK, Z (2)
- Size: 30–100 μm			
- 1–6 furrows and pores			
	Starchless	Lycopersicum peruvianum (Solanaceae): Z (1)	- *Hedera helix* (Araliaceae): PK, Z (1)
		Lamiaceae: PK, Z (1, 2, 3)	- *Borago officinalis* (Boraginaceae): PK, Z (1)
		Myrtaceae: PK, Z (1)	- Caprifoliaceae: PK, Z (1)
		Scrophulariaceae: PK, Z (1)	- Asteraceae: PK, Z (1, 2, 3)
		*Acanthus mollis* (Acanthaceae): PK, Z (2)	- *Canna indica* (Cannaceae): PK, Z (2)
		*Bryonia dioica* (Cucurbitaceae): PK, Z (2)	- *Tulipa gesneriana* (Liliaceae): PK, Z (2)
		Liliaceae sp.: PK, Z (2, 3)	
		*Mercurialis annua* (Euphorbiaceae): PK but A (2)	
**Recalcitrant pollen:**	Starchy	*Cucumis melo* (Cucurbitaceae): PK, Z (2)	Amaranthaceae: PK, Z (1)
- Desiccation sensitive		*Cucurbita pepo* (Cucurbitaceae): PK, Z (3)	Alismataceae: PK, Z (1)
- [H_2_O] dispersal: >30%		*Pistacia vera* (Anacardiaceae): A (1)	Poaceae: A (1, 2, 3)
- Size: 15–30/70–150 μm		*Plantago* sp.: PK, A (1)	*Opuntia ficus-indica* (Cactaceae): PK, Z (2)
- 0–12 (or more) pores		*Portulaca tuberosa* (Portulacaceae) PK Z (2)	*Spinacia oleracea* (Chenopodiaceae): A (1)
- No furrows		Parietaria judaica (Urticaceae) A (1)	
		Juglandaceae pp A (2)	
	Starchless	- *Laurus nobilis* (Lauraceae): PK, Z (2)	- *Cereus* sp. (Cactaceae): PK, Z (2)
		- Malvaceae: PK, Z (1, 2, 3)	- Caryophillaceae: PK, Z (1, 2)
		- *Colchicum autumnale* (Liliaceae): PK, Z (2)	
		- *Crocus sativus* (Iridaceae): PK, Z (2)	
		- *Loroglossum hircinum* (Orchidaceae): Z (1)	
		- *Ruellia prostrata* (Acanthaceae): PK, Z (1)	

*Pollenkitt (PK) is typically present in zoophilous species (Z) and is generally absent in anemophilous species (A), with the exception of some secondary anemophilous species (e.g., *Olea europaea*). Pollen sizes: (1) 15–50 μm; (2) 50–100 μm; (3) 100–150 μm. Species of the same family may have orthodox or recalcitrant pollen as in the case of *Acanthus* and *Ruellia*, and starchy or starchless pollen as *Atropa belladonna* and *Lycopersicum peruvianum* and Orchidaceae (Davis, 1966; Franchi et al., 1996; Nepi et al., 2001). The poricidal anthers of *Erica arborea* release pollen when shaken by air currents and animals*.

It is important for several reasons to improve our understanding of how DA and DT, and ultimately pollen viability and longevity are controlled. Firstly, pollen sterility induced by abiotic stresses is an agricultural problem affecting productivity of many crop species, including cereals (Powell et al., 2012). Secondly, climate change will have a significant impact on reproductive behavior of many food crops. Extremes in temperature and rainfall patterns will have a particularly large impact on pollen production and pollination capacity (Hedhly et al., 2009; Hatfield and Prueger, 2015; Mercuri et al., 2016; Urbanowicz et al., 2018). Thirdly, plant hybridization technologies often require storage of pollen grains from varieties that do not have matching flowering times, or require cross-pollination between plants that are normally self-pollinating. The aim of this paper is to give an overview about DA and DT in pollen and to instigate further research into the physiological, molecular and genetic aspects of DA and DT in plants. We have attached a glossary explaining the terminology used in this paper (Supplementary Table S1) to support those readers who are not familiar with pollen morphology.

## Analogies in DA Between Seed and Pollen: Does Pollen DA Exist?

By definition, developmental arrest (DA) is a biological term used to indicate how an entire organism, or a well-defined part of an organism, stops metabolic activity, cell divisions, growth and development in order to passively survive adverse environmental conditions. The mechanism of surviving adverse environmental conditions in an arrested state exists in most living organisms (Footitt and Cohn, 2001). DA can occur for reproductive and non-reproductive parts or the whole organism (prokaryotes, fungi, animals, plants) (Footitt and Cohn, 1995). In plants, whole-plant (vegetative) desiccation tolerance is only common in bryophytes, and rare in ferns and angiosperms where DA is used to protect reproductive parts such as spores, seeds and pollen grains (Alpert, 2005, 2006; Proctor et al., 2007).

In higher plants, seed and pollen both develop inside – and are dispersed from – involucre reproductive sporophytic structures: the anther in the case of pollen and the ovary in the case of the ovule (after fertilization, the ovary develops into the fruit while the ovule develops into the seed). At maturity, both pollen and seeds are dispersed in the environment in an arrested state (Franchi et al., 2011). There is a distinction between DA and quiescence. DA – also called dormancy – is a state where both primary metabolism and development are arrested, while in the case of quiescence only primary metabolism is arrested. Induction of dormancy in seeds inside the fruit is genetically programmed and development does not simply resume when environmental conditions return to normal. Release of dormancy only occurs under certain environmental conditions (light, temperature and stratification). The potential of the embryo to grow and develop can be repressed by signals from other parts of the seed, such as the seed coat (dicots) or the endosperm (monocots) (Foley, 2001). The plant hormone abscisic acid (ABA) plays an important role in controlling seed dormancy. ABA levels are high in dormant seeds and decrease upon imbibition. In barley, the ABA catabolic enzyme ABA 8′-hydroxylase is expressed in the protective coleorhiza tissue of the embryo root and plays a role in dormancy release(Kermode, 2005; Millar et al., 2006). ABA also plays a role in seed maturation and induction of desiccation tolerance. Desiccation involves accumulation of protective proteins such as dehydrin and Late Embryogenesis Abundant proteins (LEA), as well as various sugars and amino acids to substitute water loss and to stabilize membranes (Finch-Savage, 2003; Sreenivasulu et al., 2010). Plant LEA-like proteins are widespread and have also been found in animal species, indicating that their role in desiccation tolerance has been conserved during evolution (Browne et al., 2002).

While seeds are complex multi-cellular structures consisting of multiple specialized tissues, pollen grains are only small bi- or tri-cellular organisms. After meiosis, each young microspore normally undergoes two mitotic divisions. The first mitotic division produces the vegetative cell that hosts a smaller generative cell. In the second mitotic division, the generative cell divides again to produce the two sperm cells which are responsible for the double fertilization process in the ovary (Lord and Russell, 2002). In about 70% of plant species, the second mitotic division occurs *after* pollination and pollen germination, while in the remaining 30% of plant species both mitotic divisions occur *before* pollen dispersal to produce tri-cellular pollen. Some plant species can produce both pollen types (Lora et al., 2012; Williams et al., 2014). Orthodox pollen is most often dispersed as bi-cellular pollen in a dehydrated state, while recalcitrant pollen has a higher water content and has often completed both mitotic divisions (Williams and Brown, 2018; Williams and Reese, 2019). Tri-cellular pollen has completed the entire pollen developmental process and is ready to pollinate. This is the case for cleistogamic self-pollinating plant species. Bi-cellular pollen is immature and loses water upon dehiscence in the atmosphere. This pollen is rehydrated when it is captured by the stigma of a receptor plant before the second asymmetric mitotic division occurs (Nepi et al., 2009; Von Aderkas et al., 2018; Williams and Brown, 2018). The interruption in pollen development observed for bi-cellular pollen is similar to a true DA situation because the cell cycle is arrested. In recent years, genetic research has started to shed light on the control of cell cycle progress and cell divisions in pollen development (Durbarry et al., 2005; Oh et al., 2011; Twell, 2011; Velappan et al., 2017). Knowledge about the genes involved in controlling pollen DA may further our understanding of how the process is controlled. The developmental cycle of both bi- and tri-cellular pollen is also interrupted at the stage of pollen tube germination. Pollen germination depends on favorable interactions between pollen grains and stigma. Some genes involved in controlling pollen germination have in recent years also been identified (Liu et al., 2013; Ju et al., 2016). Control of pollen germination in plants is complex, requiring not only rehydration but also signaling events from the stigma to determine compatibility of pollen germination (Rea and Nasrallah, 2008; Dresselhaus et al., 2011; Dresselhaus and Franklin-Tong, 2013). Some genes involved in pollen-stigma interactions and pollen rehydration were recently identified (Gao et al., 2016; Li et al., 2017). It is clear that pollen development can be arrested during pollen presentation, dispersal and the pollination stage. Both seeds and pollen display DA, but the way DA manifests itself is as different as the biological and developmental features of both tissues.

The correlation between DA and DT in pollen is not strict and can be influenced by the environment. In some plant species, DA can also occur at earlier stages of pollen development. The duration of angiosperm male gametophyte development can depend on atmospheric conditions and varies considerably between species (Lersten, 2008). Development can last from a few days to a few weeks in herbaceous annual and perennial plants (Pacini and Sarfatti, 1978; Alves Rodrigues et al., 2018), and up to several months in some woody gymno- and angiosperm plants (Chesnoy, 1987; Ferranti et al., 1996). Pollen development can pause at the pollen mother cell stage in some tree species (*Pseudotsuga menziesii*, *Abies pinsapo*, *Arbutus unedo*) (Singh et al., 1983; Chiarucci et al., 1993; Arista and Talavera, 1994), at the microspore stage (*Betula verrucosa*, *Rhododendron* species) (Dunbar and Rowley, 1984; Mirgorodskaya et al., 2015), or at the bi-cellular stage (*Corylus avellana*) (Frenguelli et al., 1997). A decrease in pollen volume (12%) and a change in shape (spherical to oval) has been described in *Corylus* (Frenguelli et al., 1997). The reduction in pollen volume is accompanied by appearance of small vacuoles in the cytoplasm and pro-plastids (Dunbar and Rowley, 1984; Mirgorodskaya et al., 2015). The length of pollen development is influenced by the environment and can differ during the wet and dry season of the year (e.g., *Annona squamosa*) (Alves Rodrigues et al., 2018). When DA occurs during the early stages of pollen development, it is not always associated with desiccation. This indicates that induction of DA and DT in pollen can occur independently. This makes it harder to study DA and DT in post-dispersal pollen, as dehydration and desiccation is heavily influenced by environmental variability.

## The Intricacies of Pollen DA: Control of Dehydration and Metabolism

**Figure 2:**
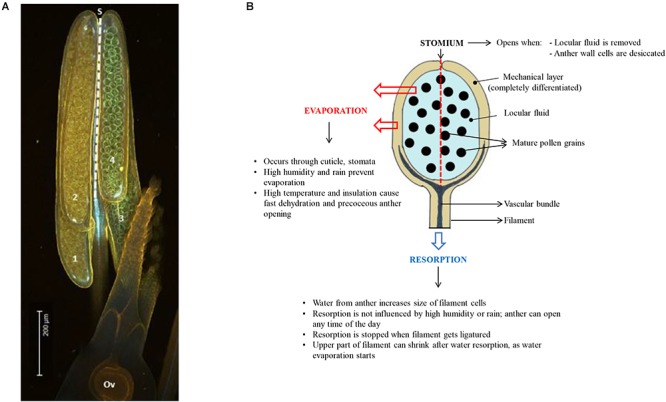
**(A)** Side view of a rice anther showing the four anther lobes (numbered 1 to 4; Ov = ovule). The dashed line indicates the position of the stomium (S) at the junction between the two anther lobes at one side of the anther (the stomium runs from the top to the base of the anther in rice). **(B)** Diagram showing a side-view of one of the 4 anther lobes, summarizing the different modes of anther dehydration that lead to pollen desiccation and DA. The stomium is located at the back of the anther lobe (dashed line). Water from the locule can evaporate through the anther wall, or it can be resorbed by the anther filament. Efficiency of evaporation depends on environmental conditions, while resorption is much less affected by the environment.

Developmental arrest in pollen is associated with changes in primary metabolism and induction of a desiccation response (DT). Pollen water content depends on the type of pollen and pollination (recalcitrant *vs*. orthodox, bi- *vs*. tri-cellular pollen). Pollen dehydration starts before dehiscence in plants producing orthodox pollen (Firon et al., 2012) and continues after dehiscence when pollen is exposed to the environment. During development, pollen is nourished by the locule fluid that is produced and secreted by the tapetum. This nutritive fluid must disappear when pollen grains complete their development, because anther opening and pollen dispersal requires the anther wall to dry out (see below) (Pacini, 2000; Scott et al., 2004; Wilson et al., 2011; Nelson et al., 2012). In many cases the cellular water level of the pollen grains also decreases during this process (Figure 1), but the dehydration process of pollen is accelerated during early phases of presentation and pollen dispersal (Firon et al., 2012). Pollen desiccation can be precipitated by evaporation when the anther opens after opening of the flower, but when the flower opens after anther opening resorption of water by the rest of the plant becomes also critical (Figure 2). In some plant species producing recalcitrant pollen (e.g., *Cucurbita pepo* and Poaceae), floral parts are responsible for absorbing the locule fluid (Nepi and Pacini, 1993). In Poaceae and lily, water is translocated in the anther filament which extends in length before opening of the stomia (Figure 2). In general, the reabsorption of water is influenced by environmental conditions such as humidity and high temperatures (Lisci et al., 1994; Bianchini and Pacini, 1996; Carrizo-García et al., 2006; Franchi et al., 2007). Before presentation and dispersal, partial dehydration of pollen can occur as a consequence of the mother plant experiencing water stress conditions; partial dehydration of recalcitrant pollen can often be observed in drought-stressed plants (Figure 1). In some cases, pollen DA and DT can be reached during early pollen presentation (Lisci et al., 1994). There is increasing evidence that active anther and pollen dehydration is genetically controlled by the mother plant. *INDUCER OF CBF EXPRESSION 1* (*ICE1*) mutations in Arabidopsis affect stomata development in the anther wall. *ICE1* mutants cause anther indehiscence and decreased pollen viability and pollen hydration, suggesting that the anther stomata play an important role in controlling pollen hydration (Wei et al., 2018). ABA plays a role in regulating stomatal closure and the ABA biosynthesis gene NCED (9-*cis*-epoxycarotenoid-dioxygenase) of rice is expressed in stomatal guard cells (Ji et al., 2011). Drought stress conditions of the mother plant leading to ABA accumulation may therefore lead to stomatal closure, preventing premature pollen dehydration before dispersal. A critical phase in pollen dehydration and acquisition of DT is after pollen dehiscence, when the reigning environmental conditions determine speed and extend of pollen dehydration (Figure 2).

Metabolic arrest can be a direct consequence of the dehydration process. Recalcitrant pollen is dispersed in a highly hydrated state and remains metabolically active. This pollen is not resistant to rapid desiccation in air and has reduced longevity. But because this pollen is metabolically active, it can germinate very quickly upon landing on a stigma. Recalcitrant pollen is therefore more suitable for self-pollinating (autogamous) plants, where exposure of pollen to air is very short (Heslop-Harrison, 1979; Franchi et al., 2002; Aylor et al., 2005). In heterogamous plants (e.g., wind, bat, insect pollinators), exposure to air is longer, so pollen needs to acquire DT and shift to a metabolically inactive state. Desiccation tolerance requires pollen grains to synthesize a set of proteins and biochemical components to protect the cellular machinery (Hoekstra and Bruinsma, 1978; Dafni and Firmage, 2000; Hoekstra et al., 2001; Firon et al., 2012). DT has to be established quickly before loss in water content in pollen causes metabolic arrest (Scott et al., 2004; Wilson et al., 2011; Nelson et al., 2012).

## Pollen Desiccation and Its Relationship to Sporophytic Drought Response

Pollen grains store water in vacuoles and the pollen water content is generally higher than 60% during development (Pacini et al., 2011). However, the anther and pollen water content may be limited by the fact that the mother plant is experiencing water stress conditions. In mature pollen, water-containing vacuoles are absent and the water content generally decreases below 40% at presentation and dispersal. Pollen in a state of DA and DT normally have a water content below 30% (Franchi et al., 2011; Pacini et al., 2011; Firon et al., 2012). A relative water content below 70% in vegetative plant tissues is normally considered a “drought stress” condition, leading to wilting and induction of ABA synthesis (Babu et al., 1999). Pollen grains are therefore likely to encounter water stress conditions at some stage during dehydration. The question is whether they will activate a water stress response similar to the one observed in the sporophyte and whether this response will be activated autonomously – or possibly involving sporophytic signals such as ABA? Acquisition of DT in pollen shows many commonalities with a typical drought response in plants and accumulation of low molecular weight osmoprotectant molecules in particular are reminiscent of osmotic adjustment – a short-term drought survival response (Hoekstra, 2005; Firon et al., 2012; Claeys and Inze, 2013). Accumulation of protective proteins and osmolytes in pollen starts after the watery vacuoles disappear from the cytoplasm (Pacini et al., 2011). From desiccation onward, orthodox and recalcitrant pollen show quantitative and qualitative differences in osmolyte levels. This may indicate that both types of pollen have a different genetic capacity to respond to desiccation. It remains to be established whether genetic differences in drought tolerance/sensitivity of the mother plant (see below) affect DT and pollen viability.

When the water content of pollen grains decreases, soluble non-reducing sugars, amino acids, soluble and insoluble pectins and other protective molecules are accumulating (Firon et al., 2012). Polyol osmolytes play a role in controlling protein folding and stability (Khan et al., 2010), but direct evidence for their presence in pollen is still missing. Polyol/monosaccharide transporters (PLT) that can import polyols into pollen have been identified and are induced by osmotic stresses, but they may play a role in importing sugars from the stigma to stimulate pollen tube growth (Klepek et al., 2005; Slewinski, 2011; Tian et al., 2017). Starch reserves accumulated during pollen development are hydrolyzed into soluble carbohydrates and there is a good correlation between sugar metabolic enzyme activities and their substrate levels (Carrizo-García et al., 2013). Mature pollen grains contain large amounts of sucrose and especially proline (Hoekstra and van Roekel, 1988; Wada et al., 1998; Hoekstra, 2002). Proline is an osmolyte that accumulates under a variety of osmotic stresses (Ashraf and Foolad, 2007; Kavi Kishor and Sreenivasulu, 2014). ABA plays an important role in drought stress response, but is also involved in various aspects of anther and pollen development, including programmed cell death of the tapetum and maturation of the anther wall (Dai et al., 2018; Kovaleva et al., 2018; Rezaul et al., 2019). ABA accumulation in pollen precedes proline accumulation (Chibi et al., 1995). In some bryophytes, ABA has been shown to be implicated in establishing desiccation tolerance (Bhyan et al., 2012; Ghosh et al., 2016).

During pollen maturation watery vacuoles are replaced by small vesicles containing low molecular weight carbohydrates with osmotic activity (Hoekstra and van Roekel, 1988; Pacini et al., 2006). The hydrolysis of starch is strongly correlated with pollen dehydration and the acquisition of DT in species with starchless pollen (Carrizo-García et al., 2013). Accumulation of soluble carbohydrates such as sucrose plays a role in membrane stabilization and protection against osmotic stress (Speranza et al., 1997; Hoekstra, 2002, 2005; Pacini et al., 2006). Variation in temperature and relative humidity in the environment affects carbohydrate accumulation, rate of dehydration and pollen viability (Guarnieri et al., 2006; Carrizo-García et al., 2010, 2014).Ripe pollen grains can be starchy or starchless, but there is always a variable proportion of soluble and insoluble cytoplasmic carbohydrates (Table 1). Sucrose and proline accumulate during pollen ripening and dehydration and disappear after rehydration (Rotsch et al., 2017). Heat stress during pollen maturation and presentation also results in glucose, fructose, sucrose and pectin accumulation (Pressman et al., 2002, 2006). The dehydration process causes an increase in dehydration stress related proteins such as osmotins to protect membranes and cytoplasm. Aquaporin water channels in the anther wall are induced to function in anther dehydration (Bots et al., 2005a,b). In pollen grains, aquaporins play a role in mobilizing soluble carbohydrates, proline and interconverting enzymes between the vegetative and generative cells. This is important for maintaining pollen viability in response to environmental conditions (Kononowicz et al., 1992; Bots et al., 2005b; Anil Kumar et al., 2015; Pérez Di Giorgio, 2016). Similar to drought and other abiotic stresses, protection against reactive oxygen species (ROS) is activated and mitochondrial activity is decreased. In mature wheat pollen, the cytoskeleton of the vegetative cell is highly organized, showing intracellular movement of organelles directed toward the pollen pore. Dehydration rapidly stops organelle movement, suggesting actin filaments of the cytoskeleton are disrupted (Heslop-Harrison and Heslop-Harrison, 1992; Heslop-Harrison et al., 1997). The induction of LEA proteins, sugars and amino acid synthesis observed in pollen grains are associated with the induction of vegetative desiccation tolerance in early land plants (Wolkers et al., 2001; Proctor et al., 2007). It is therefore likely that higher plant male gametophytes at dispersal, even though they are reduced to only two or three cells, still maintain a capacity to respond to water stress conditions. But, being heterotrophic, their capacity and dependency on the mother plant to mount an autonomous water stress response remains to be investigated.

## Pollen Desiccation and Pollen Viability

Abiotic stresses affect pollen development at all stages (Firon et al., 2012; Bokszczanin et al., 2013; Müller and Rieu, 2016; Carrizo-Garcia et al., 2017). One stage of high sensitivity is the young microspore stage, where several abiotic stresses cause tapetal dysfunction and premature abortion of pollen development. This affects self-pollinating species in particular, including important monocot and dicot crop species (Saini, 1997; Aloni et al., 2001; Pressman et al., 2002; Powell et al., 2012; Dolferus et al., 2013; De Storme and Geelen, 2014). Stress conditions can cause asynchrony later during development, affecting the number of viable pollen (Carrizo-Garcia et al., 2017), and pollen dehiscence at anthesis can also be impaired (Ribaut et al., 1996; Subedi et al., 1998; Briggs et al., 1999a,b; Jagadish et al., 2007; Chakrabarti et al., 2011; Smith and Zhao, 2016).

After dispersal, pollen longevity depends on the degree of DA and dehydration of the pollen grains, and these factors are strongly influenced by environmental conditions (Hoekstra, 2005). Because of their small size and cell number, water homeostasis of pollen grains is very easily influenced by the environment. This can act in two directions. High humidity can rehydrate desiccated pollen, decreasing longevity. Or, drought, high or low temperatures can cause further dehydration of hydrated or partially dehydrated pollen grains, causing them to eventually reach DA and improving viability. This feature may provide pollen with an adaptive advantage, as pollen viability and pollination can be postponed to sometime later when weather conditions have improved (Zonia and Munnik, 2004; Snider and Oosterhuis, 2011; Snider et al., 2011a,b).

At low levels of hydration the pollen grains enter a typical anhydrobiotic state: the cellular content becomes “glassy,” mobility of molecules and organelles is slowed down and metabolic activity becomes imperceptible (Buitink and Leprince, 2004). Reaching a glassy cellular and intracellular homeostasis is a widespread mechanism of desiccation tolerance in living organisms and is used in both angiosperm seeds and pollen grains to survive in a dry state (Farrant, 2000; Oliver et al., 2000; Leprince and Buitink, 2008; Gaff and Oliver, 2013). The glassy state is obtained when levels of water, compatible solutes (sugars and amino acids) and stabilizing proteins (dehydrin, LEA) in the pollen grains reach the right balance. However, this balance is strongly influenced by external humidity and temperature conditions (Hoekstra, 2002; Hinojosa et al., 2018). It is questionable whether pollen from all plant species can reach the “glassy” state, because it depends on synthesis of biochemical components (sugars, amino acids, proteins) that are also known to accumulate in vegetative plant parts under abiotic stress conditions. But there is genetic variability, both between and within plant species, to achieve this response (Claeys and Inze, 2013) and the question is whether this genetic variability in stress responsiveness is also translated to the male gametophyte – which is genetically related to the sporophyte. Metabolite homeostasis is crucial for cellular protection and stress tolerance and metabolites in pollen are important to maintain pollen viability and fertility (Paupiere et al., 2014; Paupière et al., 2017). Tri-nucleate recalcitrant cereal pollen is short-lived (5 to 30 min in rice and wheat, respectively) and is unable to survive longer exposures to air (D’Souza, 1970; De Vries, 1971; Hoekstra and Bruinsma, 1978; Fu et al., 2001; Wang et al., 2002). At dispersal, recalcitrant pollen does not normally reach a state of complete desiccation but pollen can dehydrate to some extend during exposure to air. Water loss to a minimum of 20–25% moisture content causes a complete loss of pollen viability; high humidity conditions can come to the rescue and extend longevity (Hoekstra, 2002). However, when low temperature conditions occur, even recalcitrant pollen can reach a glassy cytoplasm state, provided that the right level of partial dehydration and the associated osmolyte accumulation occur in the pollen grains. This property of cold temperatures has been exploited to extend pollen viability for preservation under cryogenic storage. This technique can be exploited in hybrid breeding, where pollen storage is often required to overcome differences in flowering time between plant varieties (Dafni and Firmage, 2000; Fu et al., 2001; Whitford et al., 2013; Dixon et al., 2018). In wheat, a cleistogamic self-pollinating plant species, it has been shown that the out-crossing frequency increases dramatically in field crops experiencing drought conditions (Bingham, 1966). This may indicate that some recalcitrant pollen may possess or acquire improved viability under drought conditions to increase cross-pollination potential, but it also raises questions about the extent to which abiotic stresses may cause pollen selection for stress-tolerant pollen to produce offspring. The genetic control of abiotic stress tolerance in pollen is still largely unexplored. Recent evidence shows that cyclic nucleotide-gated channels (CGNC16) may play a role in determining heat tolerance and pollen viability (Tunc-Ozdemir et al., 2013; Rahmati Ishka et al., 2018).

## Functions of the Pollen and Anther Wall in DA and DT

**Figure 3:**
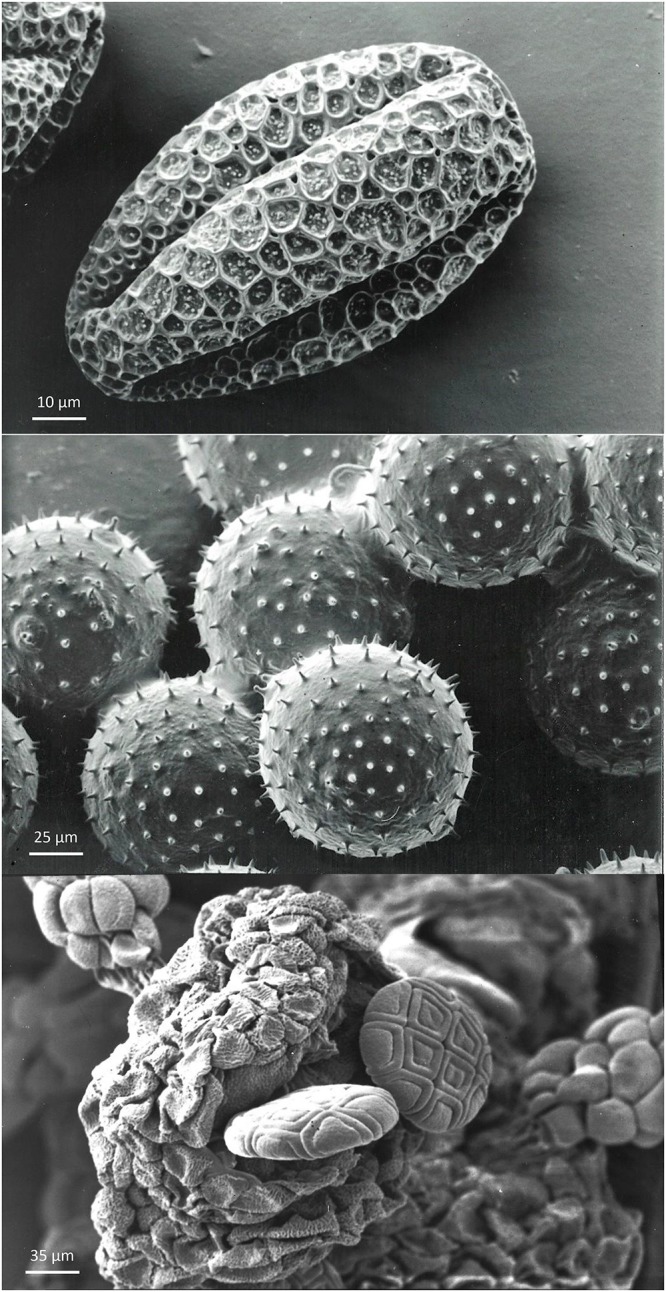
Examples of morpho-physiological pollen biodiversity. **Top:** orthodox pollen from *Citrullus vulgaris*, with the presence of furrows. These furrows facilitate variation in shape and volume of pollen in response to hydration level. Grains are spherical during development and become oval during partial dehydration before dispersal. Upon rehydration on the stigma, grains will become spherical again. **Middle:** recalcitrant *Cucurbita pepo* pollen grains without furrows. The shape of this pollen is preserved during dehydration before dispersal and rehydration on the stigma, but the volume varies according to the level of water content. Pollen grains are attached to the anther by pollenkitt and are collected by pollinators. **Bottom:** poliad *Acacia dealbata* pollen immediately after anther opening. The compound pollen grains arranged in a poliad of sixteen individual pollen grains derived from four microspore mother cells.

Pollen is surrounded by two cell walls: the external exine and the internal intine layers. Both layers act together to resist the stresses associated with volume increases and decreases occurring during pollen growth and pollen tube emission. The degree of dehydration of pollen – and later rehydration on the stigma – is regulated by the elasticity of the exine and intine layers, as well as cytological and physiological features of the vegetative cell cytoplasm (Matamoro-Vidal et al., 2016). The term harmomegathy is used to indicate these harmonic changes where walls and cytoplasm collaborate to maintain protoplast viability, notwithstanding changes in shape and volume. This process is facilitated by furrows (colpi) in the pollen wall that facilitate the collapse and change in shape of the pollen grains after losing water (Volkova et al., 2013; Figure 3).

**Figure 4:**
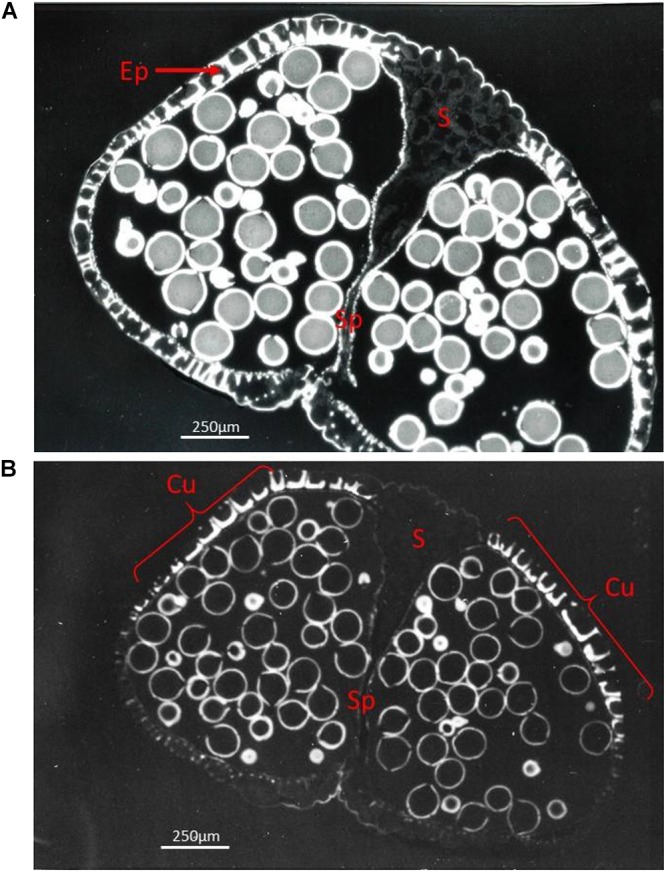
Fluorescent light pictures showing a transverse section of a ripe *Ricinus communis* bi-sporangiate anther just before dehiscence, with the stomium cells (S) still visible **(A)** and in a later stage with the stomiun cells degraded, but still not ruptured **(B)**. **(A)** The anther is surrounded by only a single layer of epidermis cells (Ep) which functions as mechanical layer. Ethidium bromide staining reveals heavily stained lignin present in the thickened cell walls of the mechanical layer positioned at the pole opposite to the stomium (S), while only weak staining is present in the stomium (S). **(B)** The Auramine O staining staining pattern shows that the cuticle (Cu) is present only in the distal and proximal poles of the anther (indicated by brackets), whilst it is practically absent from other epidermal cells. The exine of the grains is heavily stained. The cells of the stomium are now disconnected from the cells of the septum (Sp) separating the two contiguous loculi.

At the cellular level it was shown that levels of starch and soluble sugars (sucrose, glucose and fructose) change in the anther wall during maturation. Cell divisions and differentiation of the anther wall have ceased and the anther reaches its maximal size (Wilson et al., 2011). Tapetum degeneration, which starts after the first haploid mitotic division in most species, is completed when the anther has reached its maximum size. At this stage, the anther wall which is responsible for anther opening (split) and pollen release is also completely developed (Figure 4). Anther wall thickenings appear with different patterns according to anther shape and modality of anther opening (Bianchini and Pacini, 1996; Manning, 1996). Both Golgi bodies and endoplasmatic reticulum (ER) proliferate before lignin deposition starts in the anther endothecium cells; this deposition process is accompanied by a reduction in cytoplasmic volume (Whatley, 1982). All cell layers of the anther wall contain plastids, but chloroplasts with photosynthetic activity are only observed in the epidermis, endothecium and middle layer (Clément et al., 1997). In maize, chloroplasts are only present in the endothecium (Murphy et al., 2015). At the onset of anther maturation, chloroplasts differentiate into amyloplasts containing starch. The starch content in these amyloplasts is subsequently hydrolyzed and sugars are absorbed by the maturing pollen grains (Clément and Pacini, 2001).

The anther locule is filled with water and solutes for pollen nutrition. Removal of locular fluid is essential for both anther wall and pollen desiccation (Figure 2). To achieve this, the supply of water needs to be interrupted. Microscopic observations show that xylem vessels are obliterated or interrupted by fast extension growth of the anther filament (Heslop-Harrison and Heslop-Harrison, 1996), causing entry of water and nutrients in the anther to be blocked. Relocation of water and other substances occurs via the phloem vessels to other parts of the flower, e.g., nectary glands in *Petunia* (Ge et al., 2000, 2001), lodicules in grasses (Heslop-Harrison and Heslop-Harrison, 1996), or to the stamen filament and corolla (Pacini, 2000). This process is affected by environmental factors such as humidity, water stress and temperature extremes (Lalonde et al., 1997; Saini, 1997; Wilson et al., 2011).

The volume of the locular fluid increases during pollen development and is proportional to the number of pollen grains and their size, but is also determined by the shape of the pollen dispersal unit (PDU) and the arrangement of the pollen in the locule. The number of grains of a PDU can be one (e.g., grasses – Figure 1), a variable number glued together by pollenkitt, or a constant high number when single or multiple tetrads are kept together by common walls (e.g., *Acacia*; Figure 3). The number of grains composing a PDU is in many cases proportional to the number of ovules in the ovary. In grasses, pollen grains show a circular arrangement along the tapetum at the periphery of the locule, with the germination pores directed toward the tapetum plasma membrane to facilitate nutrition. An adaptation to reduce the volume of the locular fluid per anther is to have numerous smaller anthers per flower, each with fewer pollen (Ranunculaceae, Papaveraceae, Malvaceae). These anthers can open gradually according to the temperature regime (*Helleborus*) (Vesprini and Pacini, 2005), or at the same time (Malvaceae, Papaveraceae) if pollen exposure lasts for a single day only. The locular fluid volume is extremely reduced in species with compound pollen polyads (*Acacia*) and orchid pollinia (Pacini, 2010; Figure 4). In cleistogamous species the locular fluid does not disappear and pollen normally does not desiccate completely. In this case, pollen can start to emit the pollen tube inside the closed anther and perforate the anther wall to reach the stigma (cleistantheric species) (Pacini and Franchi, 1982). Under some environmental conditions, anther and pollen desiccation may not be possible. In this case, plants can be forced to become cleistogamic. Cleistogamy results in seeds with lower genetic variability in obligate cleistogamic species, but with a lower investment compared to cross-pollination (Culley and Klooster, 2007).

The products of tapetum degeneration can be totally resorbed by pollen grains, or they can be deposited on the surface of the pollen. The former is more common in plants with anemophilous pollination, while the sticky pollen surface created by pollenkitt facilitates transport by animals in zoofilous pollinators. In tetra-sporangiate anthers, the two lobes on each side of the anther are separated by small modified epidermal cells, the stomium (Figure 2, 4). The two linear stomia on each side of the anther indicate where the anther locule will split open to form a single larger locule (Keijzer, 1987). Pollenkitt is deposited on the pollen grains when the anther locules fuse and the locular fluid disappears (Pacini and Hesse, 2005). Desiccation of the anther wall cells triggers anther opening by causing the stomia cells to split (Carrizo-García et al., 2006). Enzymatic lysis starting from the loculus side causes stomia cells to detach while the outer epidermis cells stay together (Figure 4). After this process, pressure forces exerted by the mechanical layer cause the outer epidermis layer to split open, causing presentation of the pollen grains (Keijzer, 1987; Keijzer et al., 1987; Wilson et al., 2011).

In wind-pollinated species, anther opening occurs only during favorable weather conditions (warm and dry), with high solar radiation during the day time (Knox, 1979). In animal pollinated species, anther opening can occur during both day and night, depending on pollinator behavior. In night-pollinated species (bats, nocturnal birds) reabsorption of locule fluid is essential, as higher humidity during the night does not facilitate evaporation. Air humidity and thermal stresses are factors affecting the reabsorption and evaporation process during pollen ripening (Wilson et al., 2011; De Storme and Geelen, 2014). In some plant species, anthers open suddenly and pollen is released explosively. This mechanism is mediated by lignifications in the anther wall or anther filament to provide the “catapult” effect. Low air humidity triggers this explosive mechanism of pollen dispersal (Bianchini and Pacini, 1996; Franchi et al., 2007). For plant species that flower over longer periods of time, differences in metabolic activity during the season can affect pollen viability. *Parietaria* has a higher percentage of starchless pollen grains during the warmer periods compared to the cooler periods (Franchi et al., 1984). In the dioecious plant *Mercurialis annua*, which blooms all year round, pollen grains are always starchless and viability is higher than 80% throughout the year (Lisci et al., 1994).

Anther opening can be reversed in some species. This can happen during the night when humidity is higher for plants with both orthodox [*Lilium philadelphicum* (Edwards and Jordan, 1992)] and recalcitrant pollen [*Laurus nobilis* (Pacini et al., 2014)]. This modality protects pollen from precocious rehydration and is regulated by the absence of a cuticle over the anther epidermis, enabling the latter to quickly absorb and evaporate water from the environment. The corolla closes at the end of male receptivity in some hermaphrodite and monoecious species (Franchi et al., 2014).

## Pollen Dispersal, Pollination and Reversal of DA

At presentation, pollen viability is strongly influenced by environmental conditions affecting pollen water content and osmotic potential (Pacini et al., 1997; Pacini and Hesse, 2004; Pacini and Dolferus, 2016). The pollen presentation phase can vary considerably in length depending on the type of pollination: from a few seconds in self-pollinating plants (e.g., Poaceae), up to a few days in cross-pollinators (e.g., Rosaceae) where pollen sticks to the anther due to the presence of pollenkitt (Pacini and Hesse, 2005). Presentation can last for over 1 month in massulate orchids where pollen is protected inside a pollinium (Pacini, 2009). Adverse environmental conditions can cause rehydration and reversal of DA and DT, causing loss in pollen viability. The time it takes to reach a pollination target is therefore important for successful pollination. Ballistic dispersal is in some species (e.g., castor bean) used as a mechanism to speed up pollination (Bianchini and Pacini, 1996; Franchi et al., 2007). Pollen dispersal can last from a few seconds to a few hours depending on the pollen vector (air currents, animal activity). The density of pollinating targets in the area, self- or cross-compatibility of the species, the number of receptive flowers/inflorescences per plant, all determine success of pollination.

Rehydration has to occur when pollen lands on a compatible stigmatic surface. The influx of water coming from the stigma ends DA and DT. Angiosperms can have “wet” or “dry” stigmas depending on the presence of exudates on their surface. At the receptive stage the epidermis and papillae of wet stigmas secrete a fluid that helps pollen grains that land on the stigma to rehydrate. This is essential to stop pollen DA and restart metabolic activity to sustain pollen tube growth. In contrast, plants with dry stigmas have an often discontinuous (e.g., Poaceae) cuticular layer that blocks fluid secretion (Heslop-Harrison and Shivanna, 1977; Heslop-Harrison and Heslop-Harrison, 1980; Howell et al., 1993; Dickinson, 1995; Basso-Alves et al., 2011). Compatible pollen that are dispersed in a low DA state, i.e., with a water content higher than 30%, can germinate quickly and fertilize the ovule (generally ranging between a few hours up to a few days). The occurrence of DA in pollen development, the mode of pollination and the mating system of plants are closely matched features; they are also reproductive adaptation strategies for specific growth habits (Hinojosa et al., 2018).

Rehydration is quick in gymnosperms producing a pollination drop, ovular secretions that form a landing site for pollen (Mugnaini et al., 2007). In angiosperms, rehydration occurs in less than 30′ for species with recalcitrant pollen (Firon et al., 2012). In species with orthodox pollen, the process takes generally more than 15′–30′ (*Lycopersicum peruvianum*) (Pacini and Sarfatti, 1978; Franchi et al., 2002); *vice versa*, *in vitro* the pollen tube emerges after 45′ (Cresti et al., 1977). Rehydration causes the pollen volume to increase, metabolic activity starts and vacuoles re-appear in the pollen cytoplasm before pollen tube emission (Heslop-Harrison, 1987). The position of pollen tube emergence is determined by pollen-stigma spatial interactions (Heslop-Harrison et al., 1975). In higher plants, rehydration can vary between pollen grains landing contemporaneously on the stigma depending on the position of the germination pore (s) relative to the stigma surface. This can cause asynchrony in pollen tube emission and affect pollen competition (Carrizo-Garcia et al., 2017).

The pollen tube emission to fertilization phase is normally continuous and can last from less than 1 h to some days. DA can be induced during pollen tube growth, but in this case DA is associated with metabolic arrest without dehydration. This may be due to the fact that the pollen tube is growing in a protected living environment (stigma and style). This form of DA appears to play a role in compensation of asynchrony in development between the male and female gametophytes, as the female gametophyte can in some cases still be undifferentiated or in a pre-meiotic phase. When the female gametes inside the ovule reach maturity, the pollen tube resumes growth and completes fertilization. This type of DA is cytologically well documented in *Quercus suber* (Boavida et al., 1999), *Corylus heterophilla* (Liu et al., 2014) and members of the *Orchidaceae* family (Pacini, 2009; Chen and Fang, 2016).

## Conclusion: Da’s Role in Pollen Viability and Abiotic Stress Tolerance

The male gametophyte of higher plants may be reduced to only 2–3 cells, but it still holds a lot of secrets. Male gametophytes start their life as parasitic organisms; they develop in the anther locule and their development is supported by the sporophytic mother plant. But, despite their reduced size compared to ancient land plants, male gametophytes still have a short period in their life cycle where they have to function autonomously: they play a critical role in the reproductive cycle of higher plants, which requires them to travel through the atmosphere and fertilize the female gametophyte. DA and DT helps them to survive during exposure to air but this mechanism is often compromised by adverse environmental conditions (temperature stresses, humidity). It is still questionable whether pollen have a genetic capacity to deal with environmental challenges and to what extent they can achieve this. One important aspect that needs investigating in more detail is the genetic relationship between sporophyte and gametophyte: is genetic variability in abiotic stress tolerance (drought, heat, cold) of the sporophyte also translated to the gametophyte and does it contribute to a more efficient DA and DT response and better pollen viability? The answer to these questions could lead to improvements in drought-tolerance breeding and improvements in fertility of crop plants under variable climatic conditions. In wheat, osmotic adjustment has been shown to be strongly correlated with drought tolerance (Morgan, 1983; Morgan and Condon, 1986). Osmotic adjustment of pollen grains has been used to identify genetic variation for drought tolerance (Morgan and Tan, 1996; Morgan, 2000). This may indicate that osmotic adjustment and associated accumulation of osmolytes exists in pollen. The question is whether osmotic adjustment improves pollen survival capacity and the potential to induce DA and DT. Male gametophytic selection for abiotic stress tolerance in crop species has been attempted with mixed success in the past (Hormaza and Herrero, 1992; Koval, 2000; Ravikumar et al., 2003). It is possible that with improved knowledge about the genetic correlation in stress tolerance between the sporophyte and male gametophyte, and with proper control of selection conditions (temperature, humidity) this method can be made more reliable. It is also important to gain a better understanding of pollen viability and longevity and how it can be controlled. This will have positive outcomes for hybrid breeding, where increased pollen viability will improve cross-pollination efficiency and offer better options for pollen storage, e.g., cryogenic storage (Fritz and Lukaszewski, 1989; Mathad et al., 2013; Palupi et al., 2017).

## Author Contributions

Both authors have discussed and reviewed the subject and content of the manuscript, and have been involved in writing the manuscript.

## Conflict of Interest Statement

The authors declare that the research was conducted in the absence of any commercial or financial relationships that could be construed as a potential conflict of interest.
