# Supplementary material for: Pollen Developmental Arrest: Maintaining Pollen Fertility in a World With a Changing Climate
Source: Front Plant Sci. 2019 May 24;10:679. doi: 10.3389/fpls.2019.00679 (PMC6544056; doi:10.3389/fpls.2019.00679)
Supplement: Supplementary file 1 [file Table_1.pdf]

Supplementary Table S1: Glossary of terms commonly used to describe pollen morphology and development.

Term	*Definition*
• Meiocyte, pollen mother cell	Sporophytic cell in the centre of the anther that is destined to undergo meiosis and generate haploid pollen grains.
• Microspore	Alternative term used to refer to a pollen grain, but mainly used for the earlier uni-nucleate stages of pollen development. Young microspores refer to the first stage of pollen development, i.e. the cells released from the tetrad after meiosis. Microspores develop into the male gametophyte.
• Tapetum	Inner layer of the anther wall surrounding the meiocytes and loculus of the anther. Consists of secretory apoptotic cells that nourish and regulate pollen development. The tapetum degenerates, producing pollenkitt and other substances that cause pollen grains to aggregate.
• Cleistogamy/chasmogamy	Cleistogamy refers to automatic self-pollinating plants that do not open their flowers before pollen dispersal. In contrast, chasmogamy refers to plants that do open their flowers to release pollen in the environment for dispersal by animals or wind (potential cross-pollinators).
• Pollen Dispersal Unit	Pollen grains can be dispersed as single grains (monads) or as aggregates of several pollen grains kept together by viscous fluids or filaments (polyads). Tetrads derived from a single meiocyte can stay together in groups of four, united by common walls. In orchids, many packed tetrads can be arranged in different ways to form pollinia containing hundreds or thousands of pollen grains.
• Monads, polyads, pollinia	See pollen dispersal unit.
• Orthodox/recalcitrant pollen	Based on water content at dispersal, pollen grains can be classified as orthodox or recalcitrant. Orthodox pollen is desiccation resistant and has a low water content (2-20%). Recalcitrant pollen is desiccation sensitive, with water content between 20% and 50%. Orthodox and recalcitrant pollen grains both have advantages and disadvantages at pollination.
• Male germ unit	Is the association of a vegetative nucleus with a generative cell or two sperm cells to form a functional male reproductive unit in Angiosperms. The term “unit” reflects the close connection between the sperm cells and the vegetative nucleus.
• Septate/aseptate anthers	In septate anthers, in contrast to aseptate anthers, the meiocytes are separated by a wall (septum), dividing the locule in smaller compartments filled with pollen grains.
• Pollen presentation	Is the process of pollen exposure for dispersal to reach the stigma for pollination. Pollen presentation involves interaction between the anther and other floral parts. Primary presentation occurs when pollen grains are exposed in the anther. Secondary presentation involves developmental relocation of pollen from the anther to another floral organ. Pollen grains are not presented by the anther when they are launched using different mechanisms.
• Zoophilous, entomophilous and anemophilous pollen	Pollen dispersal by animals, insects and wind, respectively.
• Pollen engorgement	Pollen maturation is associated with accumulation of starch

	granules in the cytoplasm. This process is called engorgement.
• Harmomegathy	The capacity of pollen grains to change shape in response to a decrease in volume during dehydration and prior to the development arrest state. This dynamic process is controlled by the mechanical properties of the cell wall (furrows) and can be reversed by rehydration on the stigma. When pores are absent this increase and decrease in volume is due to the elasticity of exine and intine.
• Furrow, colpus	A fold region where the exine cell wall has reduced thickness, whilst intine is thicker. Furrows allow the cell wall to collapse to comply with the decrease in pollen volume during dehydration and increase volume during rehydration.
• Development arrest state	Term used to indicate the state of physiological and metabolic arrest when pollen grains reduce water content before dispersal.
• Locule, locular fluid	Central cavity in the anther where pollen grains develop. The loculus is filled with the locular fluid which is secreted by the tapetum and serves to nurture pollen. In cross section, anthers show four locules. The composition of the locule fluid changes during pollen development and before anther dehiscence the fluid is reabsorbed by the filament or other floral parts to allow pollen presentation. The locular fluid is abundant in anthers with monad and tetrad pollen, but is reduced in species with pollinia or where grains are tightly packed.
• Mechanical layer	External cell layer of the anther wall where, after tapetum degeneration, cells develop lignified wall thickenings. The mechanical layer is responsible for anther opening and pollen exposure
• Pollenkitt	Hydrophobic glue derived from the degeneration of the tapetum, composed of saturated and unsaturated lipids, carotenoids, flavonoids, proteins and carbohydrates. Pollenkitt makes grains stick to the anther, to the pollinator body and to the stigma surface.
• Pollen viability	Term used to indicate the percentage of viable pollen (i.e., able to emit pollen tubes and fertilize). Pollen viability can be assessed by hand pollination, in vitro germination and several methods evaluating physico-chemical parameters of pollen (e.g., plasma membrane intactness, the presence/abundance of some molecules or enzymes).
• Sporopollenin	Chemically and biologically resistant and elastic substance forming the building block of the exine cell wall. Sporopollenin consists of a mixture of carotene and carotenoid esters.
• Exine	External discontinuous cell wall of pollen grains. Exine is elastic, is composed of sporopollenin and has an opening called the pollen germination pore or aperture.
• Intine	Inner continuous pecto-cellulosic wall of pollen grains. The intine structure is more complex at the apertures and furrows where pollen tubes will be emitted. The intine wall becomes continuous with the pecto-cellulosic wall of the pollen tube during germination.
• Callose	Polymer of glucose residues linked together through ß-1,3-linkages. Callose is deposited during meiosis to separate the meiocytes and tetrad cells during meiosis. Callose represents a molecular filter to separate cells and is degraded by callase separated by the tapetum (ß-1,3-glucanase).
• Pollen desiccation and water content	Pollen grains desiccate before dispersal to reach equilibrium with environmental conditions. Metabolism is slowed down to better

	resist the negative effects of the environment (high or low temperature and relative humidity). Orthodox and recalcitrant pollen have different water contents at dispersal.
• Pollination drop	Liquid secreted by the ovule and exposed outside the stigma. When pollen grains land in the pollination drop, they rehydrate and germinate.
• Pollination syndrome	Term to describe the pollination traits that plants use in their natural environment to move from one flower to another, using different vectors. Plant can use abiotic (wind, water), as well as biotic (bees, birds) vectors to transfer pollen grains.
• Pollen competition	Haploid pollen grains differ in their genomic composition (recombination during meiosis) and therefore behave differently during development, pollen tube germination and in response to environmental challenges. This leads to competition between pollen grains. Pollen competition is an example of rapid Darwinian selection.
